# A comment on Farwell (2012): brain fingerprinting: a comprehensive tutorial review of detection of concealed information with event-related brain potentials

**DOI:** 10.1007/s11571-012-9217-x

**Published:** 2012-08-14

**Authors:** Ewout H. Meijer, Gershon Ben-Shakhar, Bruno Verschuere, Emanuel Donchin

**Affiliations:** 1 Department of Clinical Psychological Science, Faculty of Psychology and Neuroscience, Maastricht University, PO BOX 616, 6200 MD Maastricht, The Netherlands; 2 Department of Psychology, The Hebrew University of Jerusalem, Jerusalem, Israel; 3 Faculty of Social and Behavioural Sciences, University of Amsterdam, Amsterdam, The Netherlands; 4 Department of Psychology, Ghent University, Ghent, Belgium; 5 Department of Psychology, University of South Florida, Tampa, FL USA

**Keywords:** Concealed information test (CIT), Guilty knowledge test (GKT), Brain fingerprinting, P300

## Abstract

In a recent issue of Cognitive Neurodynamics Farwell (Cogn Neurodyn 6:115–154, 2012) published a comprehensive tutorial review of the use of Event Related Brain Potentials (ERP) in the detection of concealed information. Farwell’s review covered much of his own work employing his “brain fingerprinting” technology. All his work showed a 100 % accuracy rate in detecting concealed information. We argue in this comment that Farwell (Cogn Neurodyn 6:115–154, 2012) is misleading and misrepresents the scientific status of brain fingerprinting technology.

In a recent issue of *Cognitive Neurodynamics* Farwell (Farwell 2012) published a comprehensive tutorial review of the use of event related brain potentials (ERPs) in the detection of concealed information. Farwell’s review covered much of his own work employing his “brain fingerprinting” technology. According to the author, he and his colleagues ‘have tested brain fingerprinting technology in over 200 cases, including over a dozen scientific studies as well as individual forensic cases involving real-life crimes and other events’ (Farwell 2012 p. 135). All these studies achieved a 100 % accuracy rate in detecting concealed information. We argue in this comment that Farwell (2012) is misleading and misrepresents the scientific status of brain fingerprinting technology.

## Concealed information detection, brain fingerprinting and the P300

Brain fingerprinting is a variant of the Guilty Knowledge or Concealed Information Test (GKT/CIT; Lykken 1959, 1960). The CIT aims to determine the presence or absence of crime-related information in a suspect’s memory. It has been used with a variety of dependent measures, most notably with recordings of autonomic nervous system (ANS) measures, such as skin conductance (Ben-Shakhar and Elaad 2003). The variant of the CIT with ERPs was first investigated in the late 80ties (Farwell and Donchin 1986; Rosenfeld et al. 1988). A number of different protocols that use the P300 waveform to assess recognition of crime details have been developed, including the Farwell and Donchin (1991) protocol, which Farwell later labeled ‘brain fingerprinting’.

The brain fingerprinting approach is based on research on the P300 component, a positive brain potential that occurs between approximately 300 and 800 ms after stimulus presentation. The P300 is typically elicited in a so called oddball paradigm (Donchin 1981). In this paradigm the participants are presented with a sequence of events, with each event belonging to one of two categories. Normally events belonging to one of the categories are presented only rarely, and these events elicit a more pronounced P300. The logic underlying P300-based CIT is that as the crime relevant stimuli form a distinct-rare category relative to more frequently presented irrelevant stimuli and consequently they will elicit an enhanced P300 only in knowledgeable (guilty) participants.

## Misrepresentation

Farwell (2012) misrepresents the status of brain fingerprinting throughout his article. Using grandiloquent language [‘Prior to the invention of brain fingerprinting, the state of the art in forensic science, investigations, and criminal justice was as follows’ (Farwell 2012, p. 116)], he suggests his method revolutionized the forensic science. As explained above, brain fingerprinting is a variant of the CIT, which has been successfully applied with ANS measures for decades. Farwell erroneously claims that the ANS measures used in the CIT measure deception, while his brain fingerprinting measures recognition of crime details (Farwell 2012 p. 117). In fact, even though ANS measures and ERPs do not necessarily tap the same psychological process, both can and have been successfully used for the detection of crime details.

Considerable research since the discovery of the P300 by Sutton et al. (1965), has established that the P300 is elicited by any event that violates the subject’s expectancies. In fact, in various studies conducted in Donchin’s lab in the 70s and 80s, it was noted that occasionally some frequent events that were meaningful to some participants elicited a P300, and it was these observations that ultimately led to the P300 based CIT published in Farwell and Donchin (1991). Thus, there is no simple one-to-one relationship between the P300 and memory. Even though information stored in memory may very well cause some events to be identified as distinct and therefore elicit a P300, reducing the P300 to a simple “Aha!” response driven by ‘recognition of the relevant information contained in the probes as significant in the context of the crime’ (Farwell 2012, p. 149) is quite at variance with what is known about the P300.

The P300-MERMER, a response that Farwell claims to have discovered and for which he obtained a patent, is unlikely to solve the problem caused by the lack of a one-to-one relationship between P300 and memory. Farwell describes this P300-MERMER as the response including the P300, a late negative peak and a short-term shift in the frequency of the EEG signal. Yet the exact definition of the P300-MERMER remains vague and unclear (see Rosenfeld 2005 for an extended discussion), and as far as we can tell, Farwell never published any data in peer reviewed journals showing that the P300-MERMER has any incremental validity beyond the P300 alone. Interestingly, the University of Illinois patented the original P300 based CIT as published in Farwell and Donchin (1991). And conveniently, the ‘discovery’ and patenting of the MERMER liberates him from the constraints of this earlier patent.

## Farwell (2012) reviews of brain fingerprinting studies

Farwell (2012) reviewed and summarized 13 of his own studies (see tables 2 and 3 in Farwell (2012)). More specifically, these tables include 3 laboratory studies with a total of 73 participants and 10 field/real-life studies with a total of 132 participants. All these studies show 100 % accuracy in the detection of both the presence or absence of critical information.

However, a close inspection of the studies listed by Farwell (2012) leads to a much less optimistic view of the brain fingerprinting technology. First, of the 3 laboratory studies listed, only 1 (Farwell and Donchin 1991; Experiment 1) was published in a peer reviewed journal. Farwell lists 40 participants under this study (see Farwell 2012; table 2), but in fact it included only 20 participants, each tested twice (once in a guilty and once in an innocent condition) in a within subject design. The other two studies are merely brief conference abstracts (Farwell and Donchin 1988; Farwell and Richardson 2006a), and not articles published in peer reviewed journals. Needless to say, such conference abstracts do not contain sufficient details to judge the merits of the study, let alone allow for replication. Moreover, the 4 participants from Farwell and Donchin (1988) are reported twice as they were also included in Experiment 1 of Farwell and Donchin (1991). In sum, laboratory research on brain fingerprinting published in peer-reviewed journals amounts to a single study containing 20 participants.

Of the 10 studies that Farwell calls field/real-life studies, only 2 were published in peer reviewed journals. Farwell and Donchin (1991; Experiment 2) contained 4 participants—not 8 as listed in table 3 of Farwell (2012)—who were tested on minor real life crimes such as underage drinking (guilty condition) and on crimes committed by other participants (innocent condition). The other peer reviewed article (Farwell and Smith 2001) contained 6 participants who were tested on specific issues such as presence at a birthday party celebration in a restaurant. Three of them were tested on their own biographical data (guilty), while the other 3 were tested on information they were not aware of (innocent). All other studies listed are either conference presentations (Farwell 2009), or abstracts (Farwell 1992, 2008; Farwell and Donchin 1986; Farwell and Richardson 2006b; Farwell et al. 2011). Once again, the same 4 participants are listed twice in Table 3 of Farwell (2012), as two different studies (each time as 8 participants), once under Farwell and Donchin (1986) and then again under “real life Experiment 2″ (Farwell and Donchin 1986, 1991; Farwell 1992).

In sum, the peer reviewed data on brain fingerprinting published by Farwell and his colleagues is limited to 3 datasets with a total of 30 participants, published in two peer reviewed articles. In total, 48 correct and 6 indeterminate decisions were made in these data sets (see Table 1).

**Table 1:** Overview of studies on brain fingerprinting published in peer reviewed journals by Farwell and colleagues

Study	N participants	Correct guilty verdicts (indeterminates)	Correct innocent verdicts (indeterminates)
Laboratory studies			
Farwell and Donchin 1991, Experiment 1	20	18 (2)	17 (3)
Autobiographical studies			
Farwell and Donchin 1991, Experiment 2	4	4 (0)	3 (1)
Farwell and Smith 2001	6	3 (0)	3 (0)
Total	30	25 (2)	23 (4)

## Selection bias

Farwell’s finding of 100 % accuracy stands in sharp contrast with the available literature (See Rosenfeld 2011 for a review), and is based upon a highly selective review. Farwell (2012) posits 20 scientific standards for brain fingerprinting tests. Failure to meet these standards would explain the lower accuracy obtained by other studies. These twenty standards, however, represents merely Farwell’s subjective views, rather than a consensus within the relevant scientific community. More importantly, Farwell uses these standards selectively. He neglects to mention that studies demonstrating high detection accuracy rates also fail to meet some of these standards. In fact, his own work (Farwell and Donchin 1991) fails to meet some of these standards.

A case in point here is Farwell’s standard 4 stipulating that stimuli designated as targets should also be crime related. The original design of Farwell and Donchin (1991) embedded the crime relevant details in a classical oddball sequence among frequent irrelevant events. Some of the frequent irrelevant events were designated as targets and required a deviant button press. These targets had absolutely no relationship to the crime (‘relevant to task, not to crime’; Farwell and Donchin 1991, Table 1) and merely created a known rare category thereby providing a baseline of the subject’s normal P300. It is only under these circumstances that the elicitation of a P300 by the crime relevant events can be properly interpreted. Farwell, for some unexplained reason, now advocates the use of crime relevant events as targets, creating a different protocol. Yet he fails to discuss that Farwell and Donchin (1991) does not meet this standard.

Besides failing to meet standard 4, Farwell and Donchin (1991) also fails to meet standards 8 and 10. Yet for studies demonstrating less impressive accuracy rates, he hastens to indicate which of the standards they fail to meet. Similarly, studies demonstrating that brain fingerprinting is sensitive to countermeasures are dismissed because they do not meet all of the 20 standards, while no peer reviewed data showing that Farwell’s technique is highly resistant to countermeasures are provided.

It is important to realize that the need to publish data in refereed journals is not a merely formal requirement. Only experts in the field are capable of conducting a proper evaluation of the methods employed, the research methodology and statistical analyses used by the researcher and consequently assess the merits of the reported results. Furthermore a recent article (Simmons et al. 2011) demonstrated that inflated effect sizes are often reported even in peer reviewed journals when the methods, procedures and data analysis techniques are not properly described. All the cited Farwell studies except perhaps two did not even come close to meeting the recommendations made by Simmons et al. (2011).

## Conclusion

Many researchers—the current authors included—share a positive view towards the use of ERPs for the detection of concealed information (see also Iacono 2008). The CIT is regarded a valid paradigm (Verschuere et al. 2011), the P300 waveform is a well-established phenomenon researched in over a thousand peer reviewed publications, and many studies on the use of ERP for the detection of concealed information have been published in leading peer reviewed journals. Yet, the publication by Farwell (2012) has no place in a peer reviewed journal. By selectively dismissing relevant data, presenting conference abstracts as published data, and most worrisome, deliberately duplicating participants and studies he misrepresents the scientific status of brain fingerprinting. Thus, the review violates some of the cherished canons of science and if Dr. Farwell is, as he claims to be, a “brain fingerprinting scientist” he should feel obligated to retract the article.
